# Analysis of Clinical and Genetic Characterization of Three Ataxia–Telangiectasia Pedigrees With Novel *ATM* Gene Mutations

**DOI:** 10.3389/fped.2022.877826

**Published:** 2022-05-02

**Authors:** Peng Huang, Lu Zhang, Li Tang, Yi Ren, Hong Peng, Jie Xiong, Lingjuan Liu, Jie Xu, Yangyang Xiao, Jian Li, Dingan Mao, Liqun Liu

**Affiliations:** ^1^Department of Pediatrics, The Second Xiangya Hospital of Central South University, Changsha, China; ^2^Children's Brain Development and Brain Injury Research Office, The Second Xiangya Hospital of Central South University, Changsha, China

**Keywords:** ataxia–telangiectasia, AT, *ATM* gene, Whole-exome sequencing, Sanger sequencing

## Abstract

**Objective:**

The clinical manifestations of ataxia–telangiectasia (AT) are very complex and are easily misdiagnosed and missed. The purpose of this study was to explore the clinical characteristics and genetic features of five pediatric patients with AT from three pedigrees in china.

**Methods:**

Retrospectively collected and analyzed the clinical data and genetic testing results of five AT patients diagnosed by the Whole-exome sequencing followed by Sanger sequencing. The five patients with AT were from three pedigrees, including two female patients (case 1 and case 2) in pedigree I, one male patient (case 3) in pedigree II, and two male patients (case 4 and case 5) in pedigree III. According to the United Kingdom Association for Clinical Genomic Science Best Practice Guidelines for Variants Classification in Rare Disease 2020 to grade the genetic variants.

**Results:**

Five patients had mainly clinical presentations including unsteady gait, dysarthria, bulbar conjunctive telangiectasia, cerebellar atrophy, intellectual disability, stunted growth, increase of alpha-fetoprotein in serum, lymphopenia. Notably, one patient with classical AT presented dystonia as the first symptom. One patient had recurrent infections, five patients had serum Immunoglobulin (Ig) A deficiency, and two patients had IgG deficiency. In three pedigrees, we observed five pathogenic variants of the *ATM* gene, which were c.1339C>T (p.Arg447Ter), c.7141_7151delAATGGAAAAAT (p.Asn2381GlufsTer18), c.437_440delTCAA (p.Leu146GlnfsTer6), c.2482A>T (p.Lys828Ter), and c.5495_5496+2delAAGT (p.Glu1832GlyfsTer4). Moreover, the c.437_440delTCAA, c.2482A>T, and c.5495_5496+2delAAGT were previously unreported variants.

**Conclusions:**

Pediatric patients with classical AT may present dystonia as the main manifestation, or even a first symptom, besides typical cerebellar ataxia, bulbar conjunctive telangiectasia, etc. Crucially, we also found three novel pathogenic *ATM* gene variants (c.437_440delTCAA, c.2482A>T, and c.5495_5496+2delAAGT), expanding the *ATM* pathogenic gene mutation spectrum.

## Introduction

Ataxia telangiectasia (AT; OMIM:208900), or Louis–Bar syndrome, is a rare autosomal recessive disease with an estimated incidence of 1/100,000–1/40,000, and is caused by the biallelic mutations in the ataxia telangiectasia mutated (ATM) gene located on chromosome 11q22–23 (1). The *ATM* gene is composed of 66 exons spanning about 150kb of genomic DNA and encodes a 350-KDa protein consisting of 3,056 amino acids (2). The ATM protein is a serine/threonine-protein kinase belonging to the family of PI3K-like protein kinases and exerts a biological role by regulating the phosphorylation of many downstream proteins (3). ATM protein participates in DNA damage response, regulation of apoptosis, V(D)J recombination of immunoglobulins, T lymphocyte antigen receptor, regulation of mitochondrial functions, and Glucose metabolism (3, 4). The clinical phenotype of AT presents considerable heterogeneous and multiorgan, involving many systems such as nervous, immune, endocrine, and cardiovascular. The clinical symptoms are characterized by progressive cerebellar ataxia, oculocutaneous telangiectasia, recurrent infections caused by defective immunity, radiation sensitivity, malignancies susceptibility, retarded growth and development, and possible dyskinesia (5, 6). There are two clinical phenotypes of AT, classical AT and variant AT. Classical AT usually presents early-onset, severe clinical symptoms, and rapid progression. Cerebellar ataxia is the main clinical feature of Classical AT, often with telangiectasia, immune deficiency, and increased alpha-fetoprotein (AFP) levels. Variant AT presents late-onset, mild clinical symptoms, and slow progression, and is more common with dyskinesia, often manifesting as dystonia, myoclonus, chorea movements, tremors, and Parkinson's-like symptoms. Ataxia symptoms may be absent or may progress slowly or be only mildly involved and Telangiectasia may not occur or be unobvious. Variant AT is also more prone to tumors and metabolic diseases (7–9).

Due to the complex and diverse clinical manifestations of AT, it is easy to be misdiagnosed and missed in clinical practice. Additionally, during the disease, it is likely to be complicated by infection, pulmonary disease, malignant tumor, etc., seriously affecting the quality of life and endangering the patients' life (10). Although there is no specific treatment for AT, early detection of various complications and appropriate therapeutic intervention can improve its poor prognosis (4). Therefore, the combination of clinical manifestations and genetic testing is particularly important for early diagnosis. In this study, we performed genetic testing on three AT families, summarized clinical data, and explored the relationship between clinical phenotypes and genotypes.

## Materials and Methods

### Subjects

We retrospectively collected and descriptively analyzed medical histories, physical examinations, laboratory tests, and radiographic examinations of five AT patients diagnosed according to the European Society for Immunodeficiency Guideline (11). All of them were hospitalized in the Department of Pediatrics, Second Xiangya Hospital of Central South University from January 2015 to December 2019. The study protocols were approved by the Hospital Ethics Committee for Human Research of Second Xiangya Hospital of Central South University. The families of the patients signed the informed consent forms.

### Genetic Testing

#### Whole-Exome Sequencing (WES)

Based on the clinical suspicion, venous blood samples from the five patients and partial families of each pedigree were collected for genetic testing. Genomic DNA was extracted from venous blood to perform the WES at the genomic concentration of 50–100 ng/μl and optical density (OD) 260/280 ratios of 1.8–2.0. With an average sequence read depth of 100×, the Whole-exome capture was conducted using the Agilent SureSelect All Exon Target Enrichment System Kit (Agilent Technologies, CA, USA) based on the manufacturer's protocol (Illumina). The captured libraries were then prepared and sequenced on the Illumina NextSeq 500. We used the Burrows-Wheeler Aligner 0.6.1.11. Genome Analysis Toolkit (v1.6-7), Picard (v1.6), and SAMtools (v 0.1.18) to align the sequenced reads to the human reference sequence (GRCh37/hg19) for detecting the single nucleotide polymorphisms (SNPs) and small insertions and deletions (Indels). All variants were annotated with custom scripts. Finally, we only retained potentially deleterious variants. Low-quality genotypes (Phred-like quality scores <30) were filtered. For all retained SNPs and Indels, we primarily focused on variants within the protein-coding regions and canonical splice sites. The variants with a population frequency of >1% in public databases (the Exome Aggregation Consortium (ExAC), the Human Gene Mutation Database (HGMD), 1000 Genomes database, the Single Nucleotide Polymorphism Database (dbSNP), and Clinvar database) were excluded.

**Figure 1:**
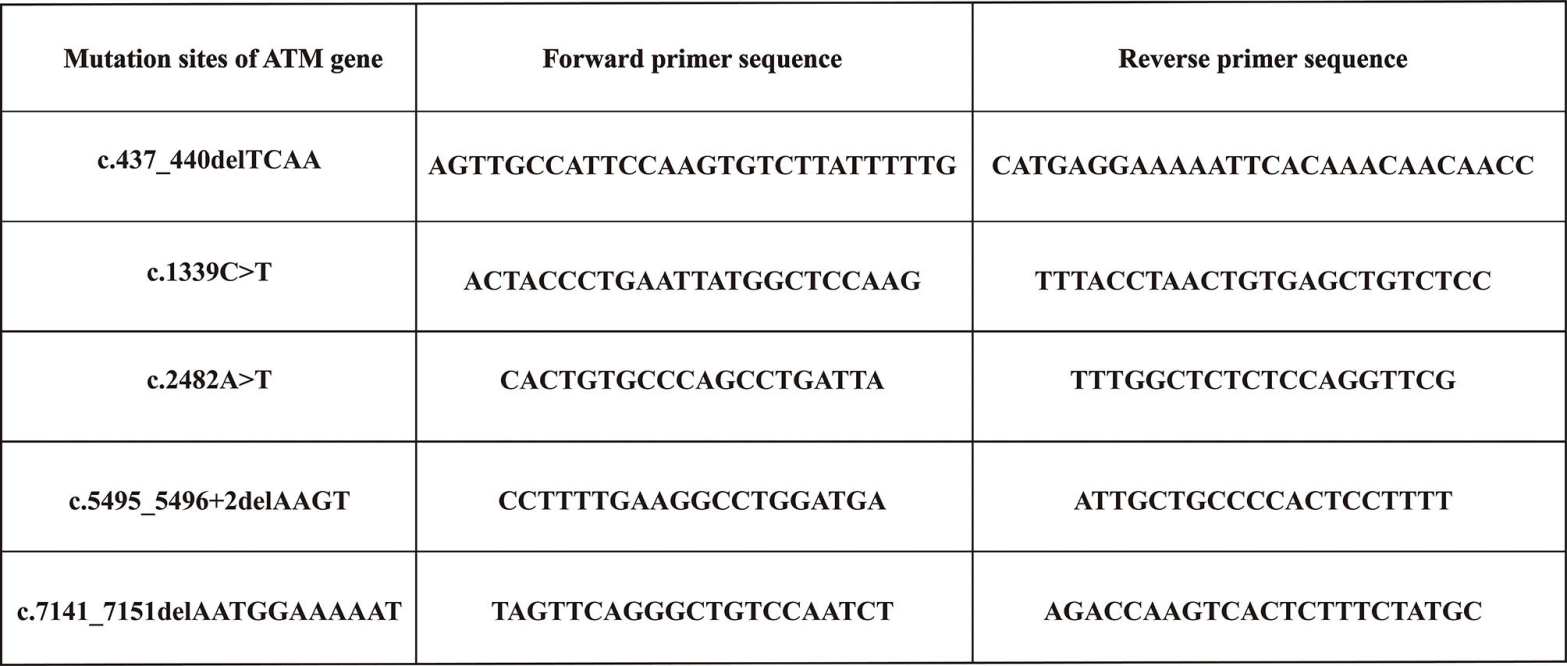
Primers sequences used to amplify the mutation sites of *ATM* gene.

Variants detected in all participants (including probands, their parents, and sibs) were verified using Sanger sequencing. The sequences corresponding to the detected variants were obtained from GenBank and used as reference strains. Then, the Primer Premier 5.0 software was used to design and synthesize specific primers for each variant. Figure 1 shows the primer sequences. The PCR-amplified products were identified, purified, recovered, and then subjected to sequence on the Sanger sequencing platform.

### Pathogenicity Analysis of Genetic Variants

Firstly, we used the bioinformatics prediction software Mutation Taster (https://www.mutationtaster.org) to predict the effect of gene mutation on protein function. Then, the pathogenicity of genetic variants was classified based on the United Kingdom Association for Clinical Genomic Science (UK-ACGS) Best Practice Guidelines for Variants Classification in Rare Disease 2020 (12), a detailed UK American College of Medical Genetics (ACMG)-based specification. The guideline also classified variants as “pathogenic”, “likely pathogenic”, “uncertain significance”, “likely benign”, and “benign” according to a series of criteria with levels of evidence defined as very strong, strong, moderate, or supporting.

## Results

### Cases Presentation

**Figure 2:**
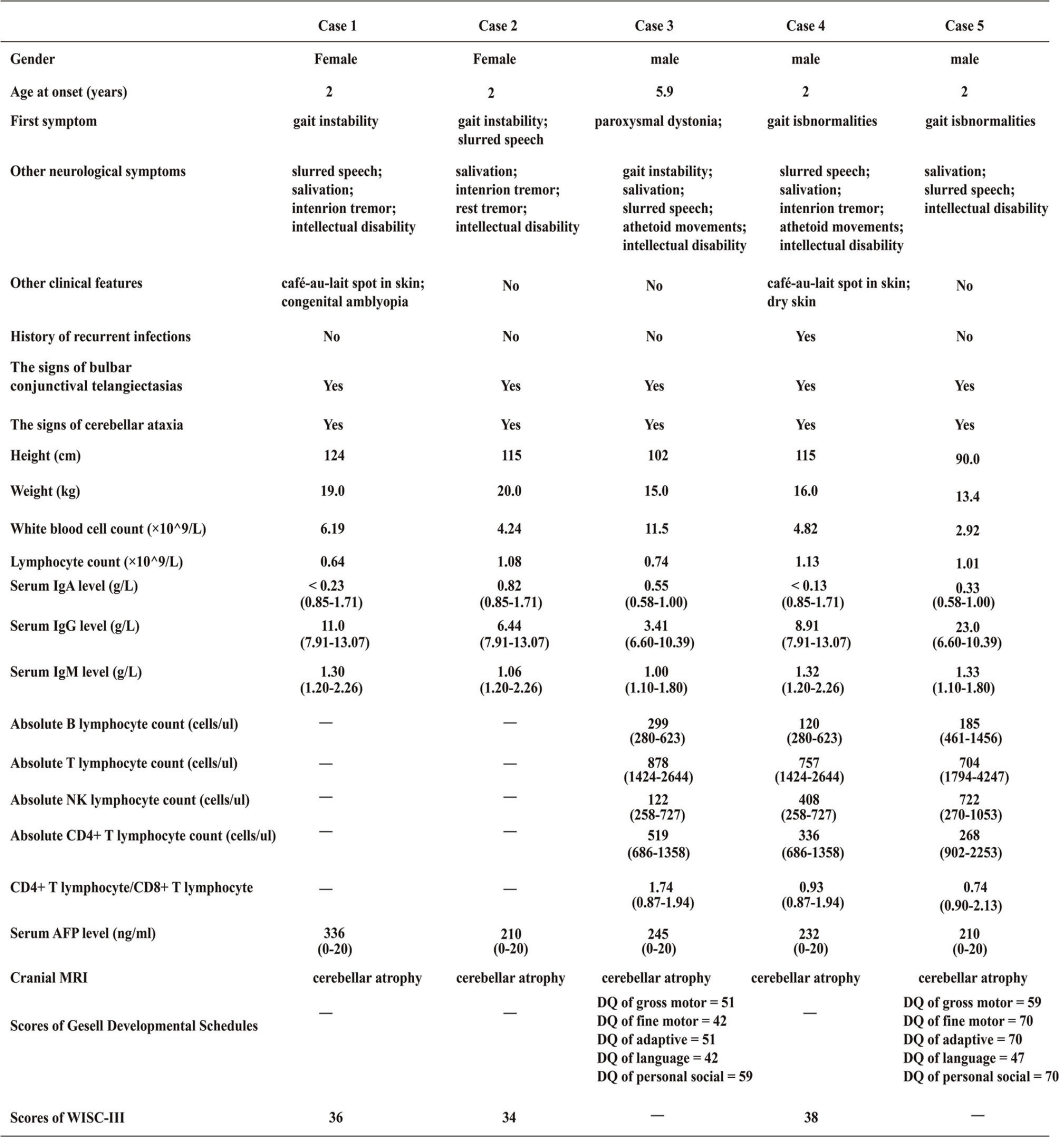
Clinical characteristics of the five patients at the time of admission. “–”, Not done; “DQ”, Developmental Quotient; “WISC-III”, Wechsler Intelligence Scale for Children Third Edition; values in parentheses represent the range of reference values.

**Figure 3:**
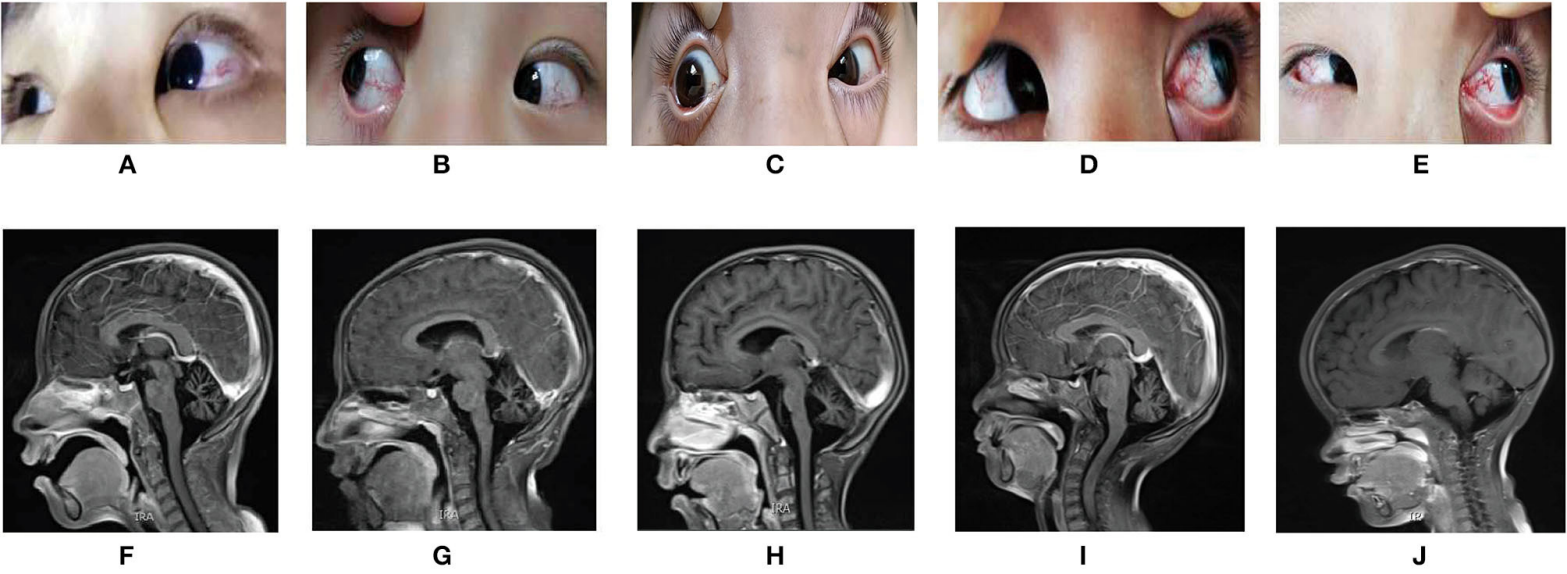
**(A–E)** showed the bulbar conjunctival telangiectasia of the five AT patients; **(F–J)** were the pictures of the Brain MRI of the five AT patients, all showed the cerebellar atrophy. **(A)** and **(F)** are from case 1, **(B)** and **(G)** are from case 2, **(C)** and **(H)** are from case 3, **(D)** and **(I)** are from case 4, **(E)** and **(J)** are from case 5.

Case 1 (The Proband 1) was an 8 years 5 months old girl born at term without complications following a normal pregnancy and was the first child of a non-consanguineous family. She was admitted to our hospital because of unsteady walking for 6 years and exacerbations for 1 week. She began to walk alone at 1-year-and-4-month-old. When nearly 2 years old, she developed gait unsteadiness, presenting wide-based gait and body sway during walking and fell easily. More and more symptoms appeared with age, including slurred speech, intention tremor, salivation, and intellectual disability (the score of WISC-III was 36, see Figure 2). She was then diagnosed with cerebral palsy at a local hospital. One week before admission, she showed fatigue in her limbs, only walking or standing with the help of others, and involuntary limb movements when she was nervous. She had no history of recurrent infections. Physical examinations on admission revealed reduced tone in four limbs. The muscle strength of her upper extremities was level 3, while it was level 4 in the lower extremities. Finger-to-nose and heel-knee-shin tests were positive. The test of rapid alternating movements showed poor coordination, the knee-jerk reflex was diminished, and the Babinski sign was negative. Laboratory findings showed that the serum AFP level was elevated, while IgA and the lymphocyte count were decreased (Figure 2). Brain magnetic resonance imaging (MRI) presented cerebellar atrophy (Figure 3). On follow-up, it was found that she had developed bulbar conjunctival telangiectasias at the age of 9 (Figure 3). When she was 10 years old, she could not walk at all except to crawl, and her slurred speech became worse.

Case 2, the younger sister of Proband 1 was a 7 years old girl born at term without complications following a normal pregnancy and was the second child of her family. She presented with similar manifestations to case 1 (see Figures 2, 3), and had no history of recurrent infections. Physical examinations on admission found the muscle strength and tone of all four limbs were normal. Finger-to-nose and heel-knee-shin tests were positive. She had difficulty in straight walking and uncoordinated rapid alternating movements. The Babinski sign was negative. During the follow-up, involuntary shaking of hands and intention tremors gradually appeared around 8 years of age. At the age of 12, her blurred speech aggravated so much that she was able to only speak 1 to 2 simple words at one time, accompanied by salivation. Meanwhile, the gait instability progressively exacerbated that body shaking during standing, falling down after walking 2–3 steps alone.

Case 3 (The Proband 2) was a 5 years 11 months old boy born at term without complications following a normal pregnancy and was the first child of a non-consanguineous family. He was admitted with paroxysmal dystonia for half-month. The main clinical manifestations frequently presented with upturned eyes, crooked mouth, distorted facial expression, clenched fists, body twisting, and frequent restlessness with no clear inducement. It only occurred when awake. Occasionally, his hands and forearms were involuntary lifted back. He could clearly speak simple words at 1-years-2-months. Gradually, he spoke a few words and had slurring of speech. He had an intellectual disability (the scores of Gesell Developmental Schedules were below normal, see Figure 2), salivation, and was misdiagnosed with epilepsy. He also had no history of recurrent infections. Physical examinations on admission showed bulbar conjunctival telangiectasia (Figure 3) and athetoid movements. The muscle tension of both lower extremities increased with cogwheel-like jerk responses. He did not cooperate with the muscle strength examination. The Babinski sign was negative. Laboratory findings showed the serum AFP increased, while IgA and lymphocyte count decreased (Figure 2). MRI presented pontine and cerebellar atrophy (Figure 3). Electroencephalogram (EEG) suggested that the θ wave activity was prominent with slow background activities during the waking period. The spike-wave discharges from the left central region occurred in the waking and sleeping periods. At the age of 7, he was re-admitted for worsening dystonia. At a follow-up, at around age 8, he developed unstable gaits presenting with wobbling and frequent falling when walking, accompanied by holding objects unsteadily. Finger-to-nose and heel-knee-shin tests were positive. He had difficulty in straight walking and needed help going upstairs and downstairs.

Case 4 (The Proband 3) represented 7 years 7 months boy born at term without complications following a normal pregnancy and was the second child of a non-consanguineous family. He was admitted for over-5-year abnormal walking postures. He could walk alone at 1 year and 4 months. When around 2 years old, he started showing abnormal walking, mainly manifesting as walking on tiptoes, wide-based gaits, waddling, head backward, and frequent falling. Often clenching fists during walking, bending inward of both upper extremities, holding objects unsteadily to drop bowls and chopsticks easily. By the age of 5, the above-mentioned symptoms on him worsened, presenting with requiring help to walk and go upstairs or downstairs, obvious wobbling when standing. He was able to speak a few easy words and had inarticulate speech. Salivation, intention tremors and intellectual disability (the score of WISC-III was 38, see Figure 2) also appeared gradually with age. Remarkably, he was susceptible to recurrent upper respiratory tract infections. Physical examinations on admission showed bulbar conjunctival telangiectasia (Figure 3) and athetoid movements. The muscle strength was level 5. Romberg test, finger-to-nose, and heel-knee-shin tests were positive. The test of rapid alternating movements showed poor coordination. He had trouble straight walking. The Babinski sign was negative. Laboratory findings showed AFP was elevated, IgA and the absolute count of T lymphocyte subsets (mainly CD4^+^ T lymphocytes) was decreased (Figure 2). Brain MRI showed cerebellar atrophy (Figure 3). There was no significant aggravation of the above symptoms during the follow-up at the age of 9.

Case 5, the younger brother of proband 3, was a 3 years 5 months boy born at term without complications following a normal pregnancy. He was the third child in his family. One year ago, he began to develop clinical features similar to case 4 (see Figures 2, 3). He had unsteady walking for 1 year, presenting moderate wobbling when walking, but not easy to fall. He could speak some simple words and had mildly slurred speech. The child also had intellectual disability (the scores of Gesell Developmental Schedules were below normal, see Figure 2) and excessive salivation. He had no recurrent infections. Physical examinations on admission found that the Romberg test and finger-to-nose test were positive. He also showed difficulty in straight walking. There was no significant aggravation of the above symptoms during the follow-up at the age of 5.

### Results of Genetic Testing

Whole exome sequencing was performed in three pedigrees, the suspected pathogenic sites were then verified by Sanger sequencing.

**Figure 4:**
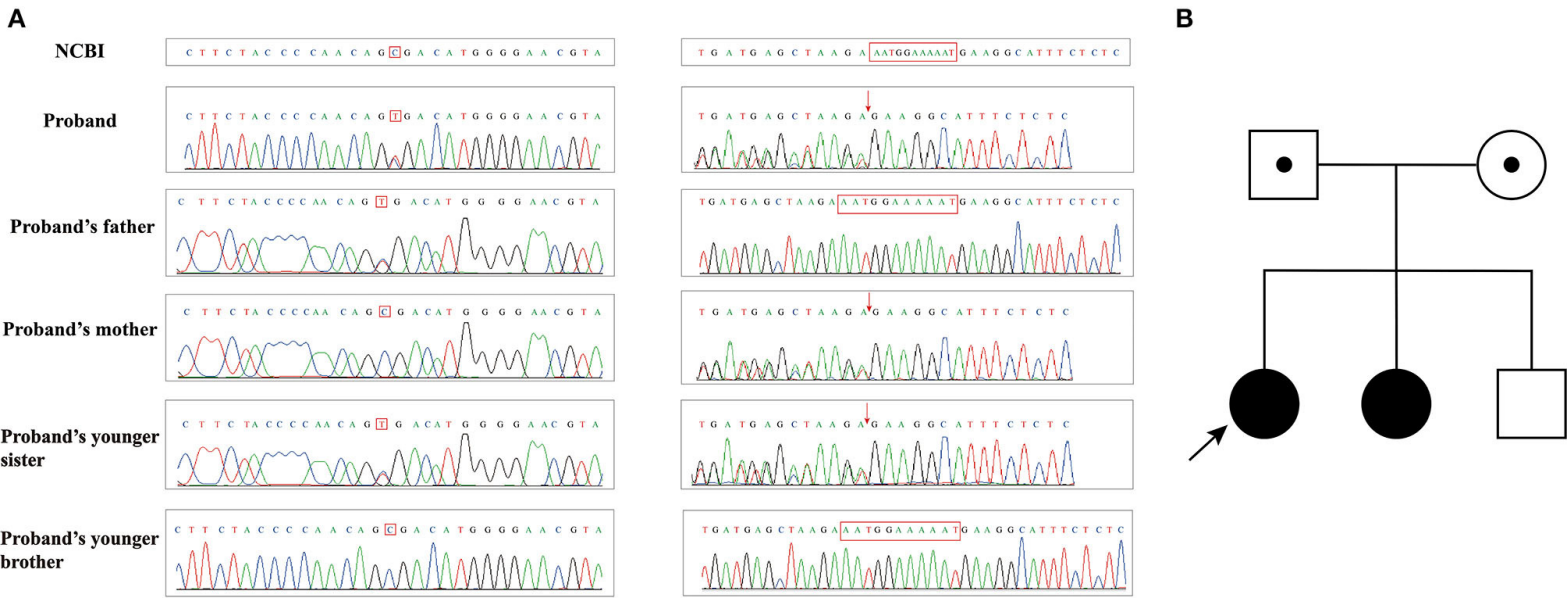
**(A)** Sanger sequencing results of family I; **(B)** Pedigree of family I.

The genetic testing results of Pedigree I showed that case 1 (Patient I-1) and case 2 (Patient I-2) harbored compound heterozygous mutations (c.1339C>T and c.7141_7151delAATGGAAAAAT) in *ATM* (NM_000051.4). The nonsense mutation in the exon 10 of *ATM* gene c.1339C>T caused the 447th amino acid to change from Arg to stop codon, resulting in premature translation termination, and was inherited from their father (Figure 4). The frameshift mutation in the exon 49 of *ATM* gene c.7141_7151delAATGGAAAAAT led to amino acid 2381 to change Asn to Glu, which caused the conversion of the 2398th amino acid to stop codon, and was inherited from their mother (Figure 4). However, their parents did not have clinical manifestations. Meanwhile, these gene mutations were not detected in their younger brother with normal a clinical phenotype.

**Figure 5:**
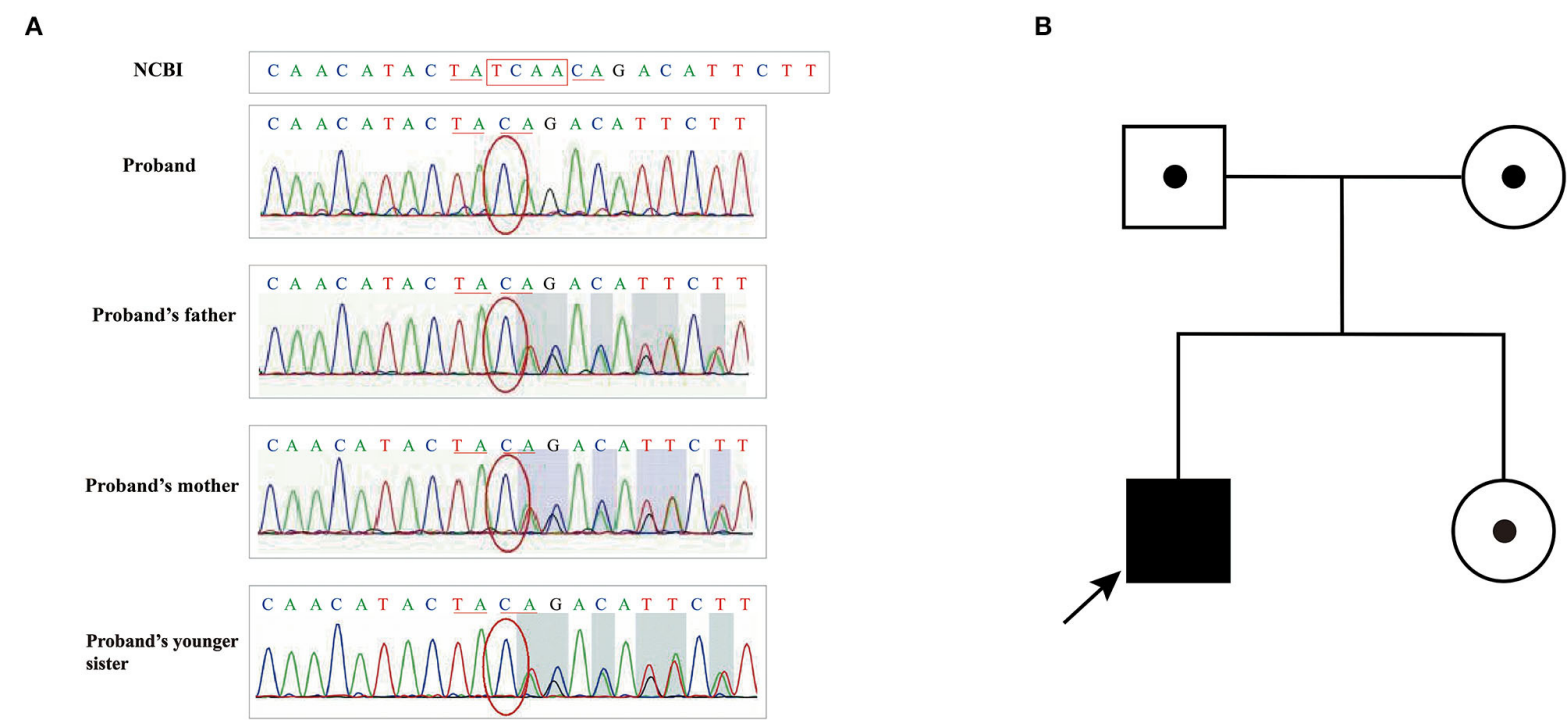
**(A)** Sanger sequencing results of family II; **(B)** Pedigree of family II.

In Pedigree II, during gene detections, it was found that the homozygous nucleotide variation of *ATM* gene (NM_000051.4) c.437_440delTCAA of case 3 (Patient II) caused the 146th amino acid change from Leu to Gln and early termination of translation synthesis at the 151st amino acid, which was inherited from his parents respectively (Figure 5). It is noteworthy that his parents and younger sister, carrying heterozygous mutations, did not have similar clinical presentations.

**Figure 6:**
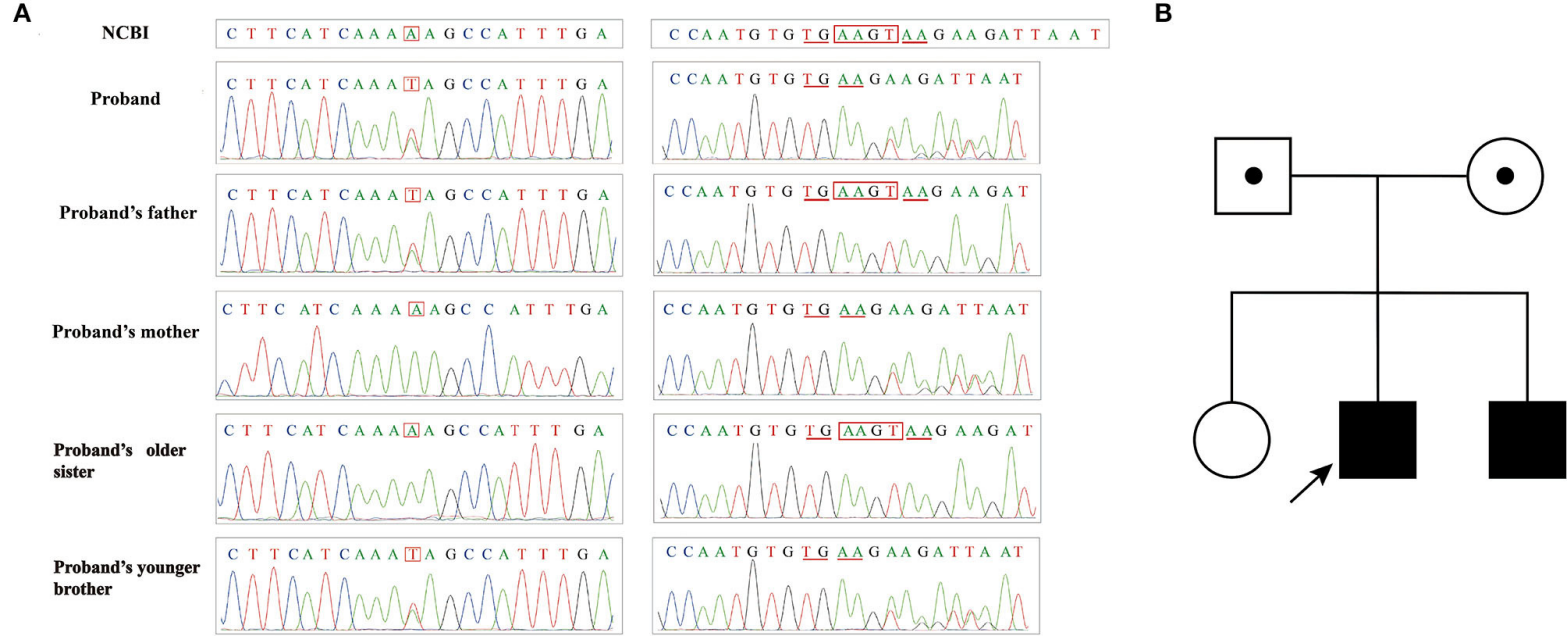
**(A)** Sanger sequencing results of family III; **(B)** Pedigree of family III.

In Pedigree III, Case 4 (Patient III-1) and Case 5 (Patient III-2) had compound heterozygous mutations (c.2482A>T and c.5495_5496+2delAAGT) in *ATM* (NM_000051.4). This nonsense mutation in the exon 17 of *ATM* gene c.2482A>T resulted in the 828th amino acid changing from Lys to stop codon, which led to premature translation termination and was inherited from their father (Figure 6). The frameshift mutation at the intron 36-exon 36 boundaries of *ATM* c.5495_5496+2delAAGT led to amino acid 1832 to change Glu to Gly and caused the transformation of the 1835th amino acid to a stop codon, which was inherited from their mother (Figure 6). Both their parents with heterozygous mutation and their sister with no mutations did not have identical clinical symptoms.

In three pedigrees, we observed the variants were completely co-segregated with the disease presenting an autosomal-recessive inheritance pattern.

### Analysis of the Pathogenicity of Gene Mutations

**Figure 7:**
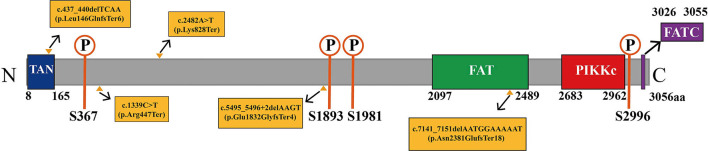
Diagram of the domain structure of ATM protein. ATM protein contains four domains, which are the TAN domain (amino acids 8–165; shown in the dark blue area), FAT domain (amino acids 2,097–2,489; shown in the green area), PIKKc domain (amino acids 2,683–2,962; shown in the red area), and FATC domain (amino acids 3,026–3,055; shown in the purple area; https://www.ncbi.nlm.nih.gov/Structure/cdd/wrpsb.cgi?INPUT_TYPE=live&SEQUENCE=NP_000042.3). ATM protein also contains multiple important autophosphorylation sites, such as S367, S1893, S1981, and S2996 (shown in the orange area). ATM is often activated through the autophosphorylation pathway to perform biological functions. The yellow triangles indicate the positions of amino acids synthesis termination corresponding to the *ATM* mutation sites identified in our patients, showing that the domains of ATM protein are damaged.

**Figure 8:**
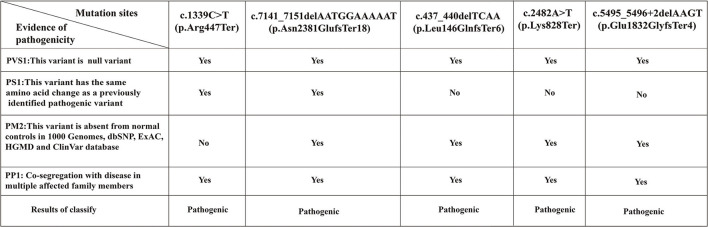
The reasons for classifying the pathogenicity of *ATM* mutation sites in 3 pedigrees. According to the UK-ACGS Best Practice Guidelines for Variant Classification in Rare Disease 2020, the variants with evidence for one PVS1 plus ≥one PS or one PVS1 plus ≥one PM are classified as Pathogenic Variants. PVS, very strong pathogenicity; PS, strong pathogenicity; PM, moderate pathogenicity; PP, supporting pathogenicity.

We analyzed the pathogenicity of each mutation in combination with the ATM protein domain. Mutations of the c.437_440delTCAA, c.1339C>T, c.2482A>T, and c.5495_5496+2delAAGT resulted in the loss of the FATC, FAT, and PIKKc domains in the *ATM* gene that are essential for kinase activity. The mutation of c.7141_7151delAATGGAAAAAT resulted in the loss of PIKKc and FATC domains. In addition, the c.437_440delTCAA, c.7141_7151delAATGGAAAAAT mutations also affected the TAN domain. Therefore, the above five gene mutations resulted in impairment or loss of domains critical to kinase activity, which could lead to complete loss of kinase activity and the disease (Figure 7). Additionally, the mutations, c.1339C>T (13) and c.7141_7151delAATGGAAAAAT (14) have been reported as pathogenic mutations. However, the remaining variants c.437_440delTCAA, c.2482A>T, and c.5495_5496+2delAAGT were previously unreported mutations. Mutation Taster software predicted five mutations to be ‘disease causing’ with a probability of 1. According to the UK-ACGS Best Practice Guidelines for Variants Classification in Rare Disease 2020, the five mutations were identified as pathogenic variants. The judgment criteria are shown in Figure 8.

## Discussions

AT is caused by homozygous or compound heterozygous mutations of the *ATM* gene. At present, more than 1,000 AT-related *ATM* gene mutations have been reported (http://www.hgmd.cf.ac.uk/ac/gene.php?gene=ATM), and their forms are complex and diverse, most are frameshift and nonsense mutations. The mutation sites cover the whole length of the gene and there are no mutation hot spots. The clinical phenotype and severity of AT depend on the residual ATM kinase activity, which is determined by the genotype. Truncating mutations, such as frameshift and nonsense variants, resulting in an almost complete loss of functional kinase activity of ATM protein, possibly leading to classical AT (7). Although missense and splicing mutations usually lead to mutated AT, the protein still exhibits a degree of kinase activity (7). Residual ATM kinase may reduce oxidative stress levels in favor of maintaining the mitochondrial function, or compensate for the loss of functional ATM proteins in other ways (e.g., modification of genes and environmental factors), which may account for the mild clinical manifestations of patients with variant AT (15, 16). In our study, all the mutations found are truncations, which by definition result in the absence of the protein product, are pathogenic, and lead to the classical AT phenotype.

It is worth noting that patient II initially presented with involuntary movements with paroxysmal dystonia as the main clinical manifestation, and the possibility of epilepsy was considered in another hospital, but no epileptic discharges were found in EEG monitoring during the same period. The brain MRI showed cerebellar atrophy at the beginning of the disease. However, it was until 3 years, after the onset of the disease that the symptoms of ataxia such as wobbliness and frequent falls appeared during walking. This differs from previously reported classical AT, as dystonia is thought to be the main feature of variant AT and is more prominent in late manifestations of classical AT (17–19). We found a typical case of AT reported by Nakayama with a compound heterozygous ATM genotype presenting with two truncating mutations, no detectable ATM protein activity in lymphoblast cell lysates, and the onset of dystonia-myoclonic jerks with ataxia, but ataxia symptoms progress slowly (16). Given the heterogeneity of clinical presentation at the onset of AT, our study also suggests that, in rare cases, ataxia may be masked by generalized dystonia, which may be the initial and most prominent neurological manifestation in patients with classical AT. The neuropathological mechanism is not fully understood, but it may be due to the severe degeneration of the basal ganglia, mainly dopaminergic neurons in the substantia nigra, affecting the integrity of the nigro-striatal pathway and leading to premature and severe dopamine uptake dysfunction (16, 20). In addition, cerebellar atrophy and cerebellar dysfunction may induce and exacerbate dystonia (21). Therefore, in patients with undiagnosed dystonia, AT should be considered, screening for AFP may help in diagnosis, and even in the absence of ataxia symptoms, the possibility of AT should be considered. In this case, genetic testing appears critical for the diagnosis of AT.

Neurological symptoms are often the initial symptoms of AT, while cerebellar ataxia is the most common. Cerebellar ataxia occurs on average at 1–2 years of age and often manifests as unsteady gait, wobbling, and easy falling when standing or walking. Most children had normal motor development in the first year after birth, followed by abnormal gaits when they learned to walk, then gradually they had difficulty walking. By the age of 10–15, with the progression of ataxia and concomitant hypotonia, eventually wheelchairs would be required (6). In our study, five patients had cerebellar ataxia, and all of them could walk normally before the onset of the disease, appearing in four patients when around 2 years old. These suggest that cerebellar ataxia is the key point for early identification of AT. The cerebellar ataxia symptoms of patients I-1 and I-2 worsened with age, and I-1 had completely lost the walking function at the age of 10, reflecting the feature of the progressive aggravation of cerebellar ataxia during the disease. However, the symptoms of cerebellar ataxia may be relatively stable between the age of 2 and 5. During the follow-up period, there was no aggravation of ataxia symptoms in patient III-2, which may gradually worsen with age. Dysarthria is the second common and persistent neurologic symptom of AT, usually appearing at the age of 1- 5 (18). The five patients in this study around 2 years of age developed dysarthria characterized by slurred or slow speech or a low pitch.

All the five patients showed bulbar conjunctival telangiectasia, which may be due to the increased expression of hypoxia-inducible factor 1 (HIF-1) after ATM protein loss, then the overexpression of HIF-1 promotes the expression of the target gene involved in angiogenesis (such as vascular endothelial growth factor), ultimately increase angiogenesis (22). Conjunctival telangiectasia often occurs after neurological symptoms, usually between the ages of 4 and 6, and then gradually spreads to the face, but may sometimes be delayed or absent (5, 23). Seven years after the onset of ataxia symptoms, patient I-1 presented bulbar conjunctival telangiectasia, indicating that for patients with ataxia without bulbar conjunctival telangiectasia, the possibility of AT should still be considered to avoid delay in diagnosis. Peripheral blood AFP is a meaningful marker of AT and is increased in most patients with AT. AFP in the five patients in this study was elevated at onset and continued to rise with age. Elevated AFP may be caused by dysfunction of AT motif binding factor-1 (ATBF1), the main repressor of *AFP* gene transcription in ATM-deficient cells (24).

Previous studies have reported that more than 70% of patients with AT had different degrees of immune dysfunction, and these patients could be combined with cellular immune deficiency and humoral immune deficiency, and infection is often the leading cause of death in AT patients (5, 25). The loss of ATM protein leads to the blocked V(D)J recombination process, which inhibits the development of the immune system and makes patients vulnerable to infection (26). Our study also showed that the five patients with AT were indeed prone to immunodeficiency, but only patient III-1 had recurrent upper respiratory tract infections, which indicated that not all immunocompromised patients would have infections. The enhancement of innate immunity may promote resistance to fight infection (27), and routine immunizations may also play a role in improving the immunity of our patients. In addition, AT patients have an increased risk of developing malignant tumors, and are prone to lymphoma, leukemia, etc., and their relatives who carry heterozygous mutations in the *ATM* gene are also prone to various tumors, such as breast cancer and gastric cancer (28). To date, no tumors have been found in the three families in this study. Considering that malignant tumors are one of the main causes of death in AT patients, close observation should be made in the later period.

The relationship between genotype and phenotype of AT is complex. The rate of ataxia progression and the timing of the onset of bulbar conjunctival telangiectasia differed between the two patients in pedigree I of the present study. Patient III-1 in pedigree III had recurrent upper respiratory tract infections, while patient III-2 did not. Pasini reported the case of a patient with AT with juvenile idiopathic arthritis (29). Amirifar reported a classical AT patient with recurrent cutaneous multiple granulomatous lesions (30). The report suggests that besides the genetic phenotype, the clinical phenotype of AT is also related to environmental factors, epigenetics, disease-modifying genes, the mutant mitochondrial DNA heteroplasmy in different tissues, etc.

## Conclusions

Pediatric patients with classical AT may present dystonia as the main manifestation, or even a first symptom, besides typical cerebellar ataxia, bulbar conjunctive telangiectasia, dysarthria, intellectual disability, stunted growth, etc. Most crucially, we found three novel pathogenic *ATM* gene mutation sites, c.437_440delTCAA, c.2482A>T, and c.5495_5496+2delAAGT, expanding the *ATM* pathogenic gene mutation spectrum.

## Data Availability Statement

The datasets presented in this article are not readily available because of privacy restrictions (the guardians of patients are reluctant to authorize the release of all the raw data of whole-exome sequencing, but agree to contribute positive sanger-sequencing results). Requests to access the datasets should be directed to the corresponding author.

## Ethics Statement

The studies involving human participants were reviewed and approved by the Hospital Ethics Committee for Human Research of Second Xiangya Hospital of Central South University. Written informed consent to participate in this study was provided by the participants' legal guardian/next of kin. Written informed consent was obtained from the individual(s), and minor(s)' legal guardian/next of kin, for the publication of any potentially identifiable images or data included in this article.

## Author Contributions

PH and LiqL designed the idea of this study. PH collected and analyzed the data and wrote this manuscript. DM and LiqL reviewed and revised this study. All authors contributed to the manuscript and approved the submitted version.

## Funding

This project was supported by China National Natural Scientific Foundation grants (Nos. 81873762 and 81501039), Science and Technology Department of Hunan Province Funds (Nos. 2022SK2032 and 2018SK2069), and Health Select Commission of Hunan Province Funds (No. B20180311).

## Conflict of Interest

The authors declare that the research was conducted in the absence of any commercial or financial relationships that could be construed as a potential conflict of interest.

## Publisher's Note

All claims expressed in this article are solely those of the authors and do not necessarily represent those of their affiliated organizations, or those of the publisher, the editors and the reviewers. Any product that may be evaluated in this article, or claim that may be made by its manufacturer, is not guaranteed or endorsed by the publisher.
